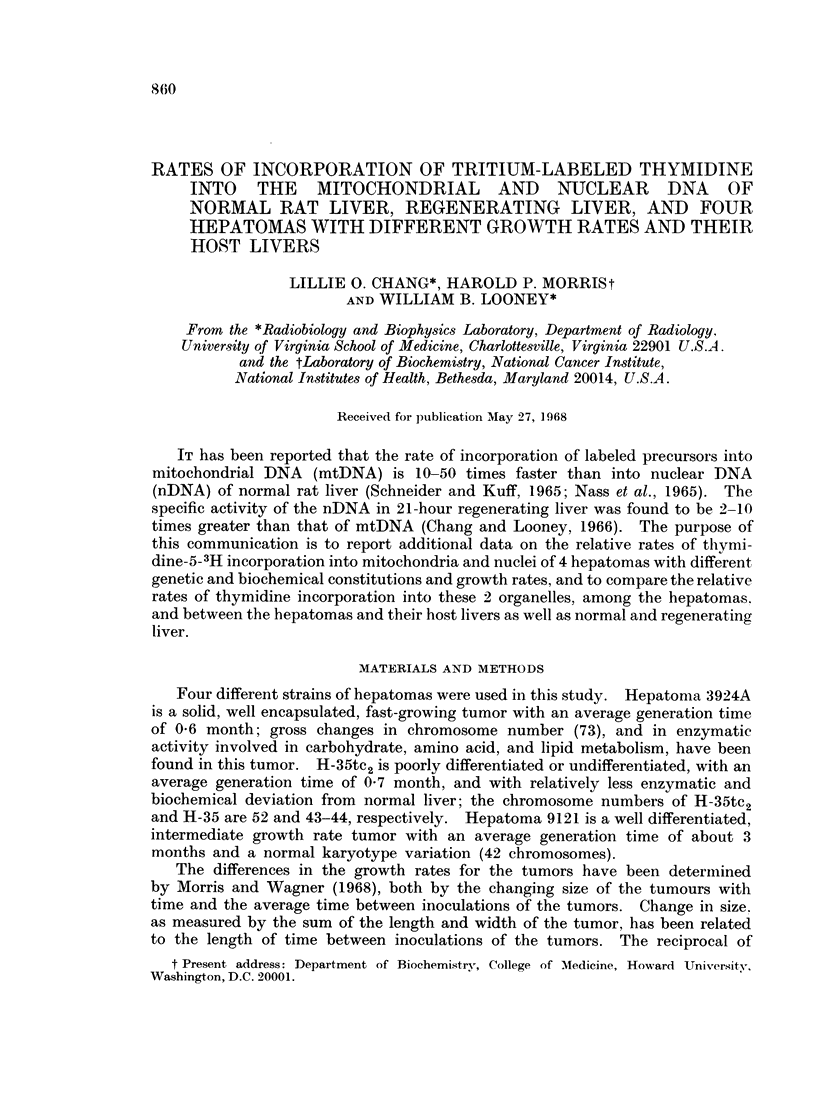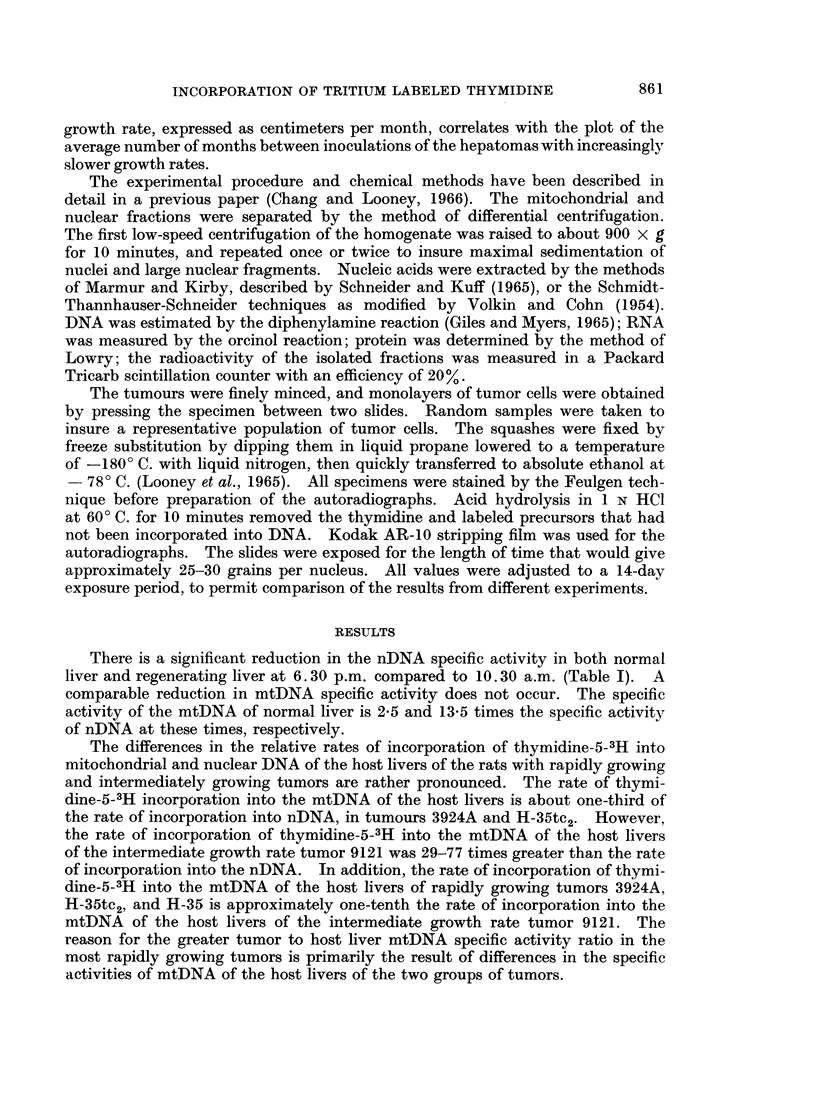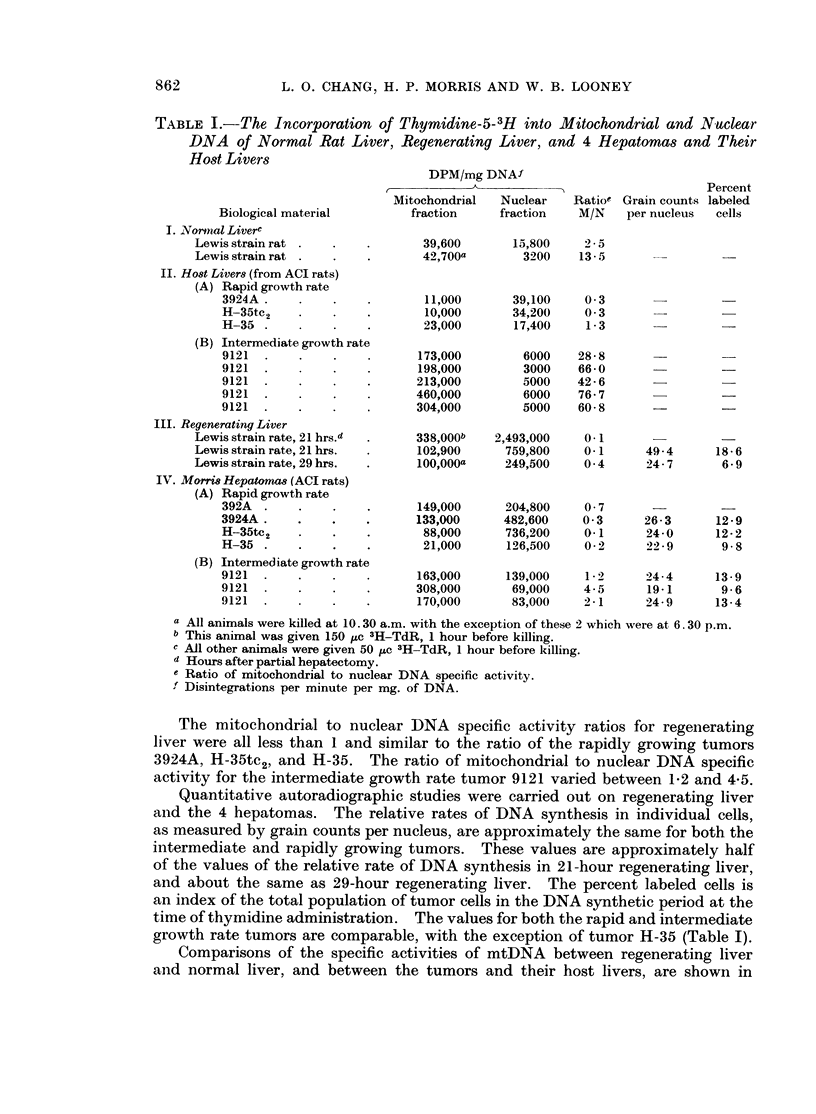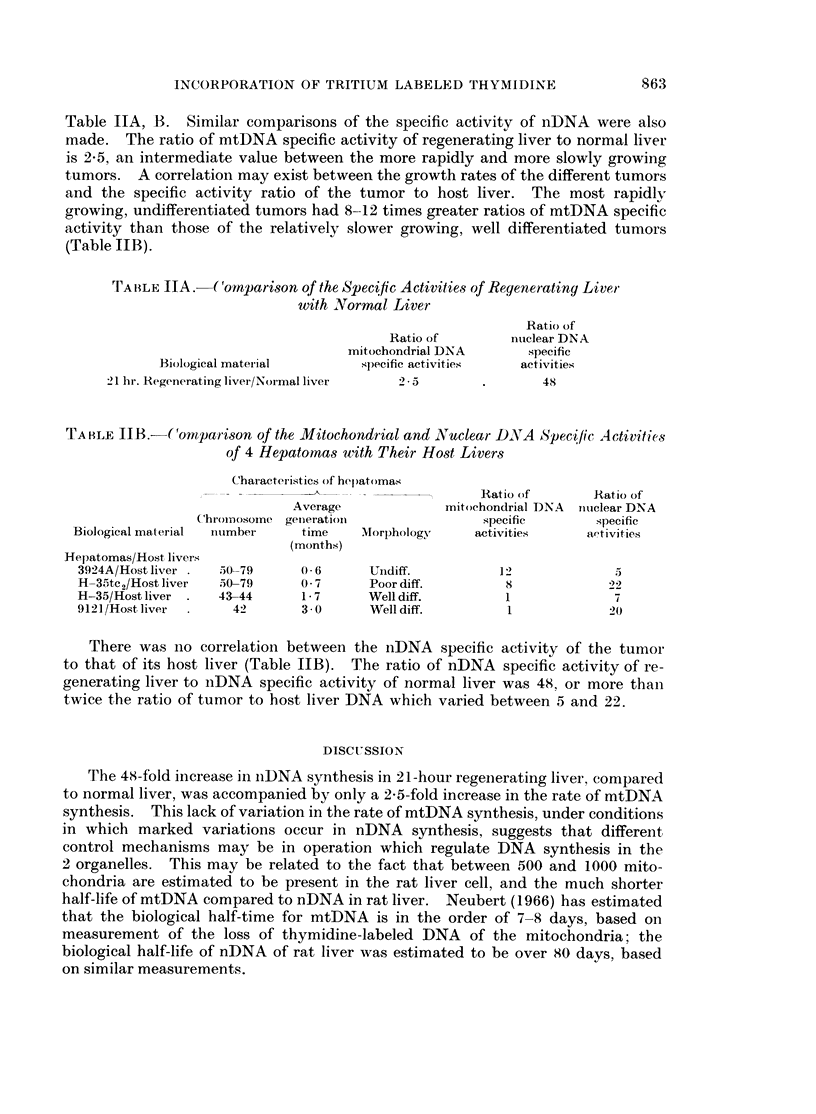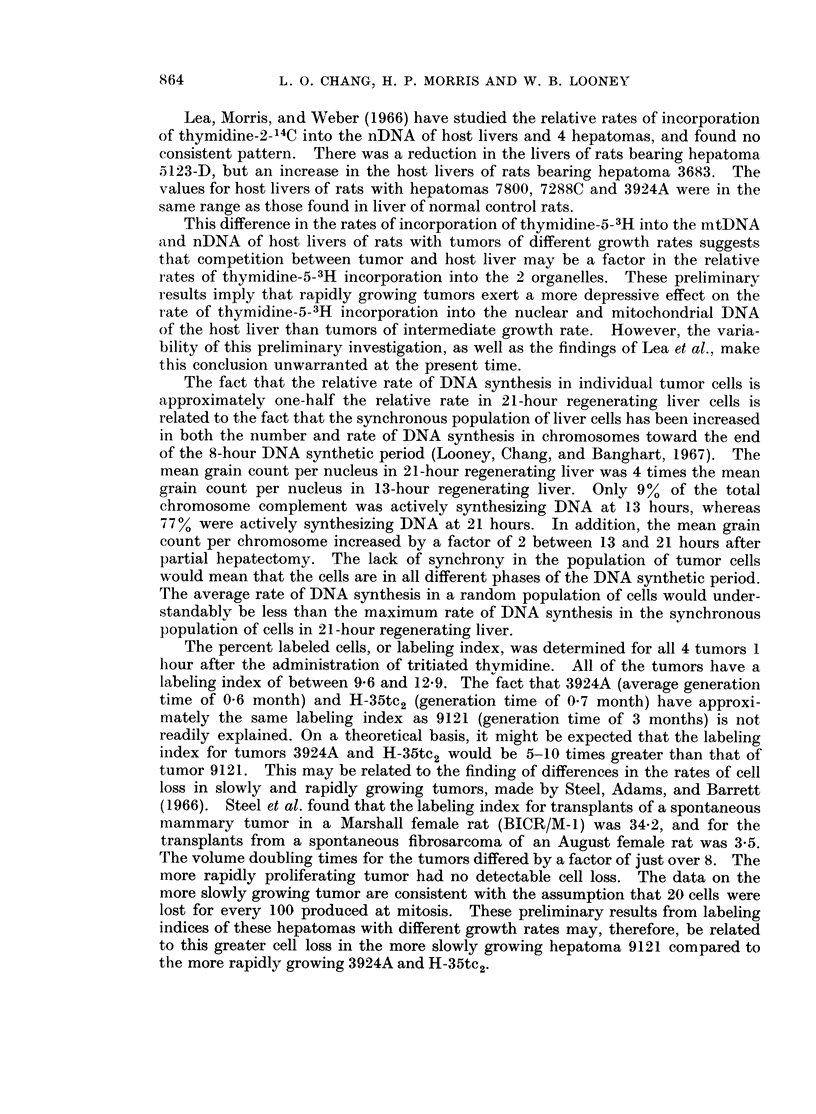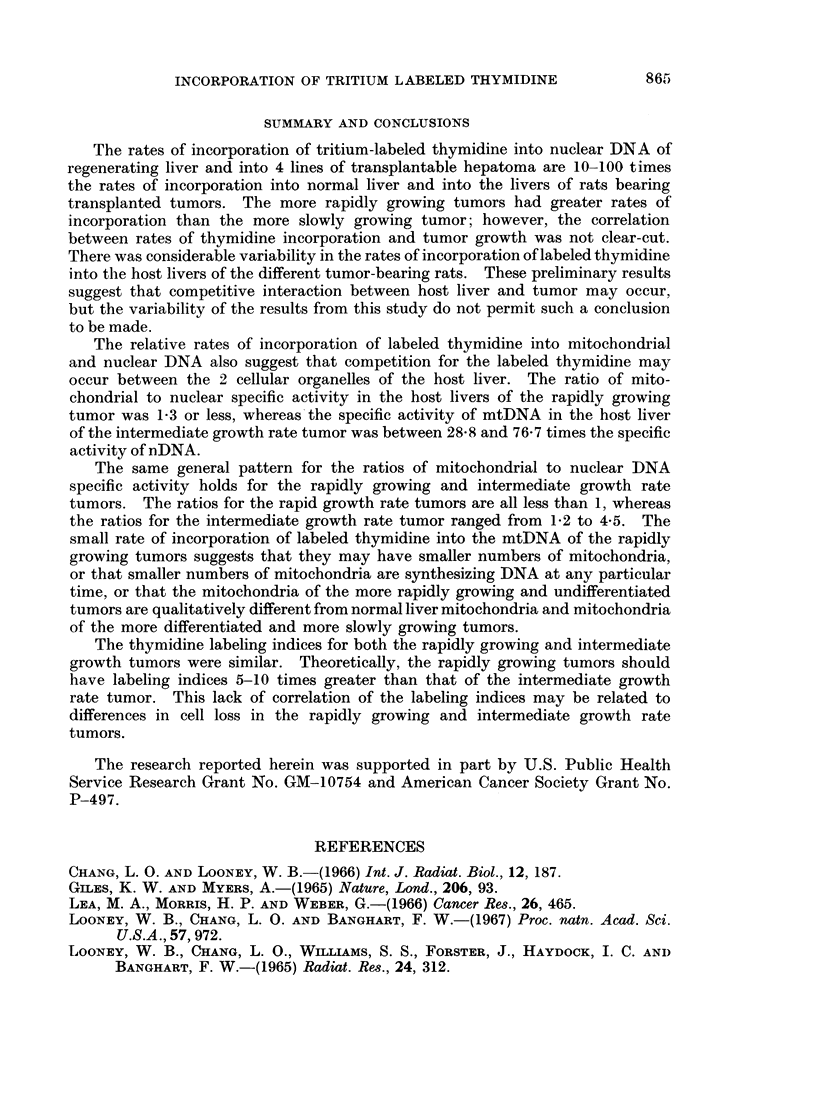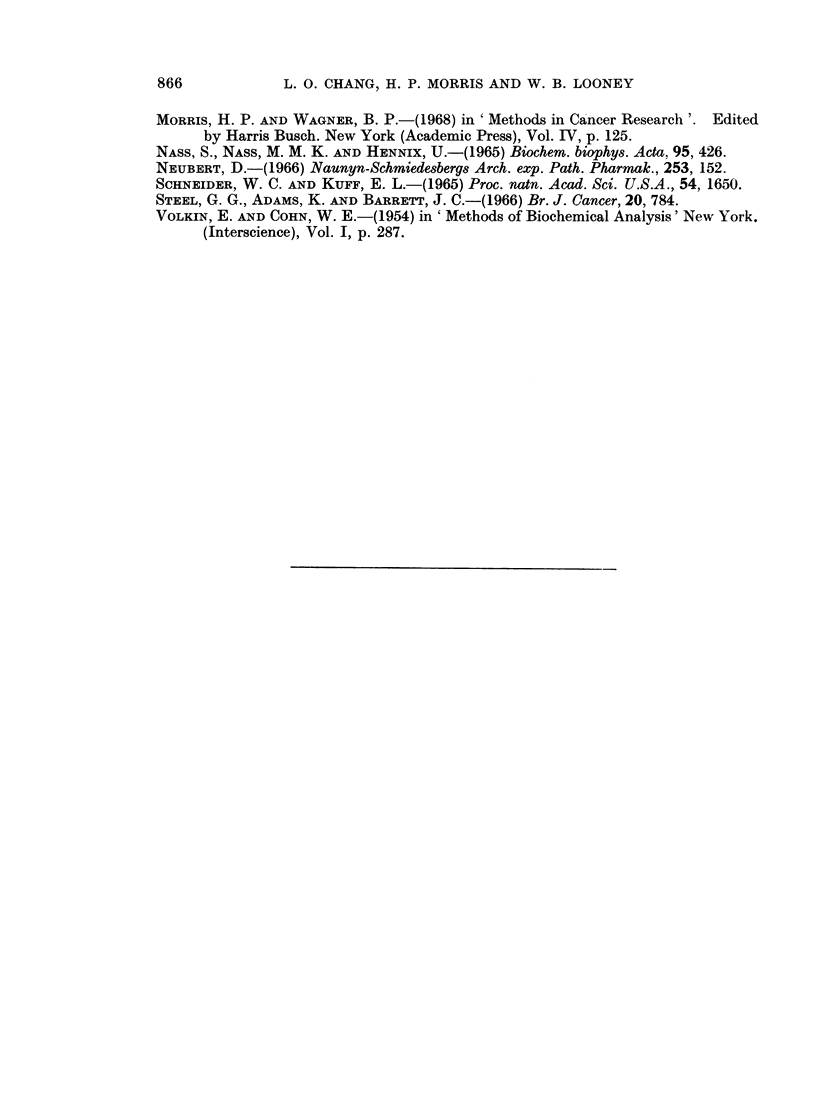# Rates of incorporation of tritium-labeled thymidine into the mitochondrial and nuclear DNA of normal rat liver, regenerating liver, and four hepatomas with different growth rates and their host livers.

**DOI:** 10.1038/bjc.1968.101

**Published:** 1968-12

**Authors:** L. O. Chang, H. P. Morris, W. B. Looney


### RATES OF INCORPORATION OF TRITIUM-LABELED THYMIDINE INTO THE MITOCHONDRIAL AND NUCLEAR DNA OF NORMAL RAT LIVER, REGENERATING LIVER, AND FOUR HEPATOMAS WITH DIFFERENT GROWTH RATES AND THEIR HOST LIVERS

LILLIE O. CHANG*, HAROLD P. MORRIS† AND WILLIAM B. LOONEY*

*From the *Radiobiology and Biophysics Laboratory, Department of Radiology, University of Virginia School of Medicine, Charlottesville, Virginia* 22901 *U.S.A. and the †Laboratory of Biochemistry, National Cancer Institute, National Institutes of Health, Bethesda, Maryland* 20014, *U.S.A.*

Received for publication May 27, 1968

It has been reported that the rate of incorporation of labeled precursors into mitochondrial DNA (mtDNA) is 10–50 times faster than into nuclear DNA (nDNA) of normal rat liver (Schneider and Kuff, 1965; Nass *et al.*, 1965). The specific activity of the nDNA in 21-hour regenerating liver was found to be 2–10 times greater than that of mtDNA (Chang and Looney, 1966). The purpose of this communication is to report additional data on the relative rates of thymidine-5-$^3$H incorporation into mitochondria and nuclei of 4 hepatomas with different genetic and biochemical constitutions and growth rates, and to compare the relative rates of thymidine incorporation into these 2 organelles, among the hepatomas, and between the hepatomas and their host livers as well as normal and regenerating liver.

### MATERIALS AND METHODS

Four different strains of hepatomas were used in this study. Hepatoma 3924A is a solid, well encapsulated, fast-growing tumor with an average generation time of 0·6 month; gross changes in chromosome number (73), and in enzymatic activity involved in carbohydrate, amino acid, and lipid metabolism, have been found in this tumor. H-35tc$_2$ is poorly differentiated or undifferentiated, with an average generation time of 0·7 month, and with relatively less enzymatic and biochemical deviation from normal liver; the chromosome numbers of H-35tc$_2$ and H-35 are 52 and 43–44, respectively. Hepatoma 9121 is a well differentiated, intermediate growth rate tumor with an average generation time of about 3 months and a normal karyotype variation (42 chromosomes).

The differences in the growth rates for the tumors have been determined by Morris and Wagner (1968), both by the changing size of the tumours with time and the average time between inoculations of the tumors. Change in size, as measured by the sum of the length and width of the tumor, has been related to the length of time between inoculations of the tumors. The reciprocal of

† Present address: Department of Biochemistry, College of Medicine, Howard University, Washington, D.C. 20001.

growth rate, expressed as centimeters per month, correlates with the plot of the average number of months between inoculations of the hepatomas with increasingly slower growth rates.

The experimental procedure and chemical methods have been described in detail in a previous paper (Chang and Looney, 1966). The mitochondrial and nuclear fractions were separated by the method of differential centrifugation. The first low-speed centrifugation of the homogenate was raised to about 900 $\times$ *g* for 10 minutes, and repeated once or twice to insure maximal sedimentation of nuclei and large nuclear fragments. Nucleic acids were extracted by the methods of Marmur and Kirby, described by Schneider and Kuff (1965), or the Schmidt-Thannhauser-Schneider techniques as modified by Volkin and Cohn (1954). DNA was estimated by the diphenylamine reaction (Giles and Myers, 1965); RNA was measured by the orcinol reaction; protein was determined by the method of Lowry; the radioactivity of the isolated fractions was measured in a Packard Tricarb scintillation counter with an efficiency of 20%.

The tumours were finely minced, and monolayers of tumor cells were obtained by pressing the specimen between two slides. Random samples were taken to insure a representative population of tumor cells. The squashes were fixed by freeze substitution by dipping them in liquid propane lowered to a temperature of —180° C. with liquid nitrogen, then quickly transferred to absolute ethanol at — 78° C. (Looney *et al.*, 1965). All specimens were stained by the Feulgen technique before preparation of the autoradiographs. Acid hydrolysis in 1 N HCl at 60° C. for 10 minutes removed the thymidine and labeled precursors that had not been incorporated into DNA. Kodak AR-10 stripping film was used for the autoradiographs. The slides were exposed for the length of time that would give approximately 25–30 grains per nucleus. All values were adjusted to a 14-day exposure period, to permit comparison of the results from different experiments.

### RESULTS

There is a significant reduction in the nDNA specific activity in both normal liver and regenerating liver at 6.30 p.m. compared to 10.30 a.m. (Table I). A comparable reduction in mtDNA specific activity does not occur. The specific activity of the mtDNA of normal liver is 2·5 and 13·5 times the specific activity of nDNA at these times, respectively.

The differences in the relative rates of incorporation of thymidine-5-$^3$H into mitochondrial and nuclear DNA of the host livers of the rats with rapidly growing and intermediately growing tumors are rather pronounced. The rate of thymidine-5-$^3$H incorporation into the mtDNA of the host livers is about one-third of the rate of incorporation into nDNA, in tumours 3924A and H-35tc$_2$. However, the rate of incorporation of thymidine-5-$^3$H into the mtDNA of the host livers of the intermediate growth rate tumor 9121 was 29–77 times greater than the rate of incorporation into the nDNA. In addition, the rate of incorporation of thymidine-5-$^3$H into the mtDNA of the host livers of rapidly growing tumors 3924A, H-35tc$_2$, and H-35 is approximately one-tenth the rate of incorporation into the mtDNA of the host livers of the intermediate growth rate tumor 9121. The reason for the greater tumor to host liver mtDNA specific activity ratio in the most rapidly growing tumors is primarily the result of differences in the specific activities of mtDNA of the host livers of the two groups of tumors.

TABLE I.—*The Incorporation of Thymidine-5-$^3H$ into Mitochondrial and Nuclear DNA of Normal Rat Liver, Regenerating Liver, and 4 Hepatomas and Their Host Livers*

| Biological material | DPM/mg DNA[f] Mitochondrial fraction | Nuclear fraction | Ratio[e] M/N | Grain counts per nucleus | Percent labeled cells |
|---|---|---|---|---|---|
| I. *Normal Liver*[c] | | | | | |
| Lewis strain rat | 39,600 | 15,800 | 2·5 | | |
| Lewis strain rat | 42,700[a] | 3200 | 13·5 | — | — |
| II. *Host Livers* (from ACI rats) | | | | | |
| (A) Rapid growth rate | | | | | |
| 3924A | 11,000 | 39,100 | 0·3 | — | — |
| H-35tc$_2$ | 10,000 | 34,200 | 0·3 | — | — |
| H-35 | 23,000 | 17,400 | 1·3 | — | — |
| (B) Intermediate growth rate | | | | | |
| 9121 | 173,000 | 6000 | 28·8 | — | — |
| 9121 | 198,000 | 3000 | 66·0 | — | — |
| 9121 | 213,000 | 5000 | 42·6 | — | — |
| 9121 | 460,000 | 6000 | 76·7 | — | — |
| 9121 | 304,000 | 5000 | 60·8 | — | — |
| III. *Regenerating Liver* | | | | | |
| Lewis strain rate, 21 hrs.[d] | 338,000[b] | 2,493,000 | 0·1 | — | — |
| Lewis strain rate, 21 hrs. | 102,900 | 759,800 | 0·1 | 49·4 | 18·6 |
| Lewis strain rate, 29 hrs. | 100,000[a] | 249,500 | 0·4 | 24·7 | 6·9 |
| IV. *Morris Hepatomas* (ACI rats) | | | | | |
| (A) Rapid growth rate | | | | | |
| 392A | 149,000 | 204,800 | 0·7 | — | — |
| 3924A | 133,000 | 482,600 | 0·3 | 26·3 | 12·9 |
| H-35tc$_2$ | 88,000 | 736,200 | 0·1 | 24·0 | 12·2 |
| H-35 | 21,000 | 126,500 | 0·2 | 22·9 | 9·8 |
| (B) Intermediate growth rate | | | | | |
| 9121 | 163,000 | 139,000 | 1·2 | 24·4 | 13·9 |
| 9121 | 308,000 | 69,000 | 4·5 | 19·1 | 9·6 |
| 9121 | 170,000 | 83,000 | 2·1 | 24·9 | 13·4 |

[a] All animals were killed at 10.30 a.m. with the exception of these 2 which were at 6.30 p.m.
[b] This animal was given 150 $\mu c$ $^3$H–TdR, 1 hour before killing.
[c] All other animals were given 50 $\mu c$ $^3$H–TdR, 1 hour before killing.
[d] Hours after partial hepatectomy.
[e] Ratio of mitochondrial to nuclear DNA specific activity.
[f] Disintegrations per minute per mg. of DNA.

The mitochondrial to nuclear DNA specific activity ratios for regenerating liver were all less than 1 and similar to the ratio of the rapidly growing tumors 3924A, H-35tc$_2$, and H-35. The ratio of mitochondrial to nuclear DNA specific activity for the intermediate growth rate tumor 9121 varied between 1·2 and 4·5.

Quantitative autoradiographic studies were carried out on regenerating liver and the 4 hepatomas. The relative rates of DNA synthesis in individual cells, as measured by grain counts per nucleus, are approximately the same for both the intermediate and rapidly growing tumors. These values are approximately half of the values of the relative rate of DNA synthesis in 21-hour regenerating liver, and about the same as 29-hour regenerating liver. The percent labeled cells is an index of the total population of tumor cells in the DNA synthetic period at the time of thymidine administration. The values for both the rapid and intermediate growth rate tumors are comparable, with the exception of tumor H-35 (Table I).

Comparisons of the specific activities of mtDNA between regenerating liver and normal liver, and between the tumors and their host livers, are shown in

Table IIA, B. Similar comparisons of the specific activity of nDNA were also made. The ratio of mtDNA specific activity of regenerating liver to normal liver is 2·5, an intermediate value between the more rapidly and more slowly growing tumors. A correlation may exist between the growth rates of the different tumors and the specific activity ratio of the tumor to host liver. The most rapidly growing, undifferentiated tumors had 8–12 times greater ratios of mtDNA specific activity than those of the relatively slower growing, well differentiated tumors (Table IIB).

TABLE IIA.—*Comparison of the Specific Activities of Regenerating Liver with Normal Liver*

| Biological material | Ratio of mitochondrial DNA specific activities | Ratio of nuclear DNA specific activities |
|---|---|---|
| 21 hr. Regenerating liver/Normal liver | 2·5 | 48 |

TABLE IIB.—*Comparison of the Mitochondrial and Nuclear DNA Specific Activities of 4 Hepatomas with Their Host Livers*

| Biological material | Characteristics of hepatomas: Chromosome number | Average generation time (months) | Morphology | Ratio of mitochondrial DNA specific activities | Ratio of nuclear DNA specific activities |
|---|---|---|---|---|---|
| Hepatomas/Host livers | | | | | |
| 3924A/Host liver | 50–79 | 0·6 | Undiff. | 12 | 5 |
| H-35tc$_2$/Host liver | 50–79 | 0·7 | Poor diff. | 8 | 22 |
| H-35/Host liver | 43–44 | 1·7 | Well diff. | 1 | 7 |
| 9121/Host liver | 42 | 3·0 | Well diff. | 1 | 20 |

There was no correlation between the nDNA specific activity of the tumor to that of its host liver (Table IIB). The ratio of nDNA specific activity of regenerating liver to nDNA specific activity of normal liver was 48, or more than twice the ratio of tumor to host liver DNA which varied between 5 and 22.

### DISCUSSION

The 48-fold increase in nDNA synthesis in 21-hour regenerating liver, compared to normal liver, was accompanied by only a 2·5-fold increase in the rate of mtDNA synthesis. This lack of variation in the rate of mtDNA synthesis, under conditions in which marked variations occur in nDNA synthesis, suggests that different control mechanisms may be in operation which regulate DNA synthesis in the 2 organelles. This may be related to the fact that between 500 and 1000 mitochondria are estimated to be present in the rat liver cell, and the much shorter half-life of mtDNA compared to nDNA in rat liver. Neubert (1966) has estimated that the biological half-time for mtDNA is in the order of 7–8 days, based on measurement of the loss of thymidine-labeled DNA of the mitochondria; the biological half-life of nDNA of rat liver was estimated to be over 80 days, based on similar measurements.

Lea, Morris, and Weber (1966) have studied the relative rates of incorporation of thymidine-2-$^{14}C$ into the nDNA of host livers and 4 hepatomas, and found no consistent pattern. There was a reduction in the livers of rats bearing hepatoma 5123-D, but an increase in the host livers of rats bearing hepatoma 3683. The values for host livers of rats with hepatomas 7800, 7288C and 3924A were in the same range as those found in liver of normal control rats.

This difference in the rates of incorporation of thymidine-5-$^3H$ into the mtDNA and nDNA of host livers of rats with tumors of different growth rates suggests that competition between tumor and host liver may be a factor in the relative rates of thymidine-5-$^3H$ incorporation into the 2 organelles. These preliminary results imply that rapidly growing tumors exert a more depressive effect on the rate of thymidine-5-$^3H$ incorporation into the nuclear and mitochondrial DNA of the host liver than tumors of intermediate growth rate. However, the variability of this preliminary investigation, as well as the findings of Lea *et al.*, make this conclusion unwarranted at the present time.

The fact that the relative rate of DNA synthesis in individual tumor cells is approximately one-half the relative rate in 21-hour regenerating liver cells is related to the fact that the synchronous population of liver cells has been increased in both the number and rate of DNA synthesis in chromosomes toward the end of the 8-hour DNA synthetic period (Looney, Chang, and Banghart, 1967). The mean grain count per nucleus in 21-hour regenerating liver was 4 times the mean grain count per nucleus in 13-hour regenerating liver. Only 9% of the total chromosome complement was actively synthesizing DNA at 13 hours, whereas 77% were actively synthesizing DNA at 21 hours. In addition, the mean grain count per chromosome increased by a factor of 2 between 13 and 21 hours after partial hepatectomy. The lack of synchrony in the population of tumor cells would mean that the cells are in all different phases of the DNA synthetic period. The average rate of DNA synthesis in a random population of cells would understandably be less than the maximum rate of DNA synthesis in the synchronous population of cells in 21-hour regenerating liver.

The percent labeled cells, or labeling index, was determined for all 4 tumors 1 hour after the administration of tritiated thymidine. All of the tumors have a labeling index of between 9·6 and 12·9. The fact that 3924A (average generation time of 0·6 month) and H-35tc$_2$ (generation time of 0·7 month) have approximately the same labeling index as 9121 (generation time of 3 months) is not readily explained. On a theoretical basis, it might be expected that the labeling index for tumors 3924A and H-35tc$_2$ would be 5–10 times greater than that of tumor 9121. This may be related to the finding of differences in the rates of cell loss in slowly and rapidly growing tumors, made by Steel, Adams, and Barrett (1966). Steel *et al.* found that the labeling index for transplants of a spontaneous mammary tumor in a Marshall female rat (BICR/M-1) was 34·2, and for the transplants from a spontaneous fibrosarcoma of an August female rat was 3·5. The volume doubling times for the tumors differed by a factor of just over 8. The more rapidly proliferating tumor had no detectable cell loss. The data on the more slowly growing tumor are consistent with the assumption that 20 cells were lost for every 100 produced at mitosis. These preliminary results from labeling indices of these hepatomas with different growth rates may, therefore, be related to this greater cell loss in the more slowly growing hepatoma 9121 compared to the more rapidly growing 3924A and H-35tc$_2$.

### SUMMARY AND CONCLUSIONS

The rates of incorporation of tritium-labeled thymidine into nuclear DNA of regenerating liver and into 4 lines of transplantable hepatoma are 10–100 times the rates of incorporation into normal liver and into the livers of rats bearing transplanted tumors. The more rapidly growing tumors had greater rates of incorporation than the more slowly growing tumor; however, the correlation between rates of thymidine incorporation and tumor growth was not clear-cut. There was considerable variability in the rates of incorporation of labeled thymidine into the host livers of the different tumor-bearing rats. These preliminary results suggest that competitive interaction between host liver and tumor may occur, but the variability of the results from this study do not permit such a conclusion to be made.

The relative rates of incorporation of labeled thymidine into mitochondrial and nuclear DNA also suggest that competition for the labeled thymidine may occur between the 2 cellular organelles of the host liver. The ratio of mitochondrial to nuclear specific activity in the host livers of the rapidly growing tumor was 1·3 or less, whereas the specific activity of mtDNA in the host liver of the intermediate growth rate tumor was between 28·8 and 76·7 times the specific activity of nDNA.

The same general pattern for the ratios of mitochondrial to nuclear DNA specific activity holds for the rapidly growing and intermediate growth rate tumors. The ratios for the rapid growth rate tumors are all less than 1, whereas the ratios for the intermediate growth rate tumor ranged from 1·2 to 4·5. The small rate of incorporation of labeled thymidine into the mtDNA of the rapidly growing tumors suggests that they may have smaller numbers of mitochondria, or that smaller numbers of mitochondria are synthesizing DNA at any particular time, or that the mitochondria of the more rapidly growing and undifferentiated tumors are qualitatively different from normal liver mitochondria and mitochondria of the more differentiated and more slowly growing tumors.

The thymidine labeling indices for both the rapidly growing and intermediate growth tumors were similar. Theoretically, the rapidly growing tumors should have labeling indices 5–10 times greater than that of the intermediate growth rate tumor. This lack of correlation of the labeling indices may be related to differences in cell loss in the rapidly growing and intermediate growth rate tumors.

The research reported herein was supported in part by U.S. Public Health Service Research Grant No. GM–10754 and American Cancer Society Grant No. P–497.

### REFERENCES

Chang, L. O. and Looney, W. B.—(1966) *Int. J. Radiat. Biol.*, **12**, 187.

Giles, K. W. and Myers, A.—(1965) *Nature, Lond.*, **206**, 93.

Lea, M. A., Morris, H. P. and Weber, G.—(1966) *Cancer Res.*, **26**, 465.

Looney, W. B., Chang, L. O. and Banghart, F. W.—(1967) *Proc. natn. Acad. Sci. U.S.A.*, **57**, 972.

Looney, W. B., Chang, L. O., Williams, S. S., Forster, J., Haydock, I. C. and Banghart, F. W.—(1965) *Radiat. Res.*, **24**, 312.

MORRIS, H. P. AND WAGNER, B. P.—(1968) in ' Methods in Cancer Research '. Edited by Harris Busch. New York (Academic Press), Vol. IV, p. 125.
NASS, S., NASS, M. M. K. AND HENNIX, U.—(1965) *Biochem. biophys. Acta*, **95**, 426.
NEUBERT, D.—(1966) *Naunyn-Schmiedesbergs Arch. exp. Path. Pharmak.*, **253**, 152.
SCHNEIDER, W. C. AND KUFF, E. L.—(1965) *Proc. natn. Acad. Sci. U.S.A.*, **54**, 1650.
STEEL, G. G., ADAMS, K. AND BARRETT, J. C.—(1966) *Br. J. Cancer*, **20**, 784.
VOLKIN, E. AND COHN, W. E.—(1954) in ' Methods of Biochemical Analysis ' New York. (Interscience), Vol. I, p. 287.